# Introduction to the Southern African HIV Clinicians Society harm reduction guidelines

**DOI:** 10.4102/sajhivmed.v21i1.1179

**Published:** 2020-12-17

**Authors:** Edwin Cameron

**Affiliations:** 1 Judicial Inspectorate of Correctional Services, Pretoria, South Africa

Lawyers have done much damage in South Africa. Apartheid was enforced, minutely and brutishly, through the law. Its central weapon, viciously wielded, was the criminal law.

That legacy looms large over our still-new, still-nascent constitutional democracy. It is particularly painful in the case of people who use drugs.

For more than 50 years, the criminal law has waged war on people who use drugs. The consequences have been deadly. Criminalising people who use drugs is not only vicious, as apartheid’s laws were. It is stupid, and ineffective. It does nothing to diminish the use of drugs.

Prisons rarely advance rehabilitation, and indeed prison is no place where someone dependent on drugs could hope to resolve their dependence or find ‘rehabilitation’.

Globally, leaders and institutions, including the United Nations, have conceded this. They now acknowledge that criminalising people who use drugs is not an appropriate solution and that treating them as criminals inflicts significant harm on society.

In South Africa, the debate has been muted. Current policies rely on a criminal justice response to drug use. So far, this has attracted little public criticism, although former President Motlanthe has recently added his considerable stature to the call to decriminalise drug use: he has spoken out strongly against prohibition, repression, harsh penalties and prison for drug-related offences.^1^

It is notable and timely that in June 2019 the Southern African HIV Clinicians Society (SAHCS) published a position paper supporting decriminalisation of drug use.

Like sex, the pursuit of mind-altering substances is part of being human.^2^ This seemingly radical statement is in fact an inoffensive truism. And hence, as with sex, the ‘just say no’ message is futilely misplaced in reducing transmission of human immunodeficiency virus (HIV) between people who use drugs.

Furthermore, research has established that people who use drugs are not a homogeneous group, and the drug experience is mediated by not only the pharmacology of the drug but also individual vulnerabilities and mindsets, in addition to the context in which the drugs are used.^3^

The majority of people who use drugs will not suffer harmful consequences. However, misguided application of the criminal law inflicts harm, disastrously. People who are marginalised, excluded and stigmatised are far more likely to suffer harm from their drug use, as well as become addicted to drugs.^4^

Recent years have seen an increase in the use of drugs in South Africa, as well as in the numbers of people dependent on heroin. The use of drugs like Nyaope, Whoonga and Unga is increasing, as is the injecting of heroin and stimulant drugs.^5^

This calls, imperatively, for harm reduction services, including providing sterile injecting equipment. But they are absent. This invites an increase in blood-borne infections. The prevalence of HIV amongst people who inject drugs is 21%, and the rate of hepatitis C is 55%.

The Joint United Nations Programme on HIV/AIDS (UNAIDS) warns, if South Africa does not take action now, it will never reach the 2020 fast track targets for HIV reduction that we missed.^6^

Here, history grimly repeats itself. The barriers people with and at risk of HIV and acquired immunodeficiency syndrome (AIDS) faced are strikingly similar to those people using drugs face now: fear, exclusion, stigma, prejudice, disregard of human rights, science and compassion.

Instead, there is the terribly misplaced invocation of the criminal justice system. There is an underlying and deep-rooted belief that deviance must be punished. The crude remedies that inflicted apartheid are reassigned to persecute people who use drugs (the same is true in the case of sex workers and people not conforming to traditional gender constructs). Criminalising people who use drugs is a damaging distraction from the most effective means we have to reduce the consequences of drug use: harm reduction.

Harm reduction is a practical and rights-informed approach to assist people in reducing drug-related harms and to support them in changes they seek to make.

Conceived in the 1980s, from the imperative to stop the rapid spread of HIV amongst people who use drugs, the harm reduction movement has been fostered by HIV activists and HIV clinicians.

Therefore, it is fitting that SAHCS leads. These harm reduction guidelines embody empirically indicated and rights-respecting doctrine in both decriminalising drug use and adopting best clinical practice to reduce the spread of HIV and other blood-borne viruses amongst people who use and inject drugs.

The guidelines make sound sense. Not only do they build on the statement supporting the decriminalisation of the use of certain drugs, but they also take a public health approach rather than an abstinence and recovery-focused approach. Also, they take into account the context, that is, what components drive the dependent and habitual use of drugs?

Valuable lessons we learned from the response to HIV apply to people who use drugs. Whilst people who use drugs may need health services, pathologising them inflicts significant stigma and exclusion.

We fail in our response to people who use drugs by simplistically depicting them as either prisoners or patients. They are first and foremost people, who have the constitutionally enshrined right to be treated with dignity and to receive the highest standard of care available.

The lessons are clear. Countries that have embraced the principles of harm reduction have seen a reduction in the rates of HIV infection. They have benefited from lower overdose deaths and reductions in the adverse health, social and economic consequences from the use of drugs.^7^

Of course, harm reduction is not without its critics, even within the medical community. The counter-arguments proceed principally from moral axiom. The belief that people should be abstinent from drugs is as misguided as the belief that HIV can be prevented by teaching people to abstain from sex. To deny people harm reduction services echoes the damage that refusing to make condoms available futilely inflicted.

The policy landscape in South Africa has shifted, and for the first time, harm reduction is described in the National Drug Master Plan. However, this needs to be backed by political determination. We must replace the misplaced abstinence-based approach with an approach proven to help reduce the consequences of using unregulated drugs.

Without addressing the underlying motivators, addressing drug use in isolation will seldom help.

The guidelines are, therefore, more than a simple set of algorithms or treatment regimens. They describe the reasons why people may develop problematic drug use, the systemic and contextual issues, psychosocial and biomedical intervention and evidence-based approaches for more vulnerable populations.

Even amongst clinicians, there are gaps in understanding people who use drugs and their medical needs. The guidelines are timely and well-directed, in supporting clinicians who engage with and treat people who use drugs. It is imperative that they are used and disseminated widely.

South Africa is burdened in many areas by shame, including internalised shame and stigma.^8^ These hobble the power of people to act fully as citizens; they isolate people and allow anger and fear to amass.

The notion that all people who use drugs are selfish, dishonest or powerless risks becoming self-fulfilling. These guidelines counter internalised stigma by including the voices of people who use drugs. This step is not radical. It is elementary. And essential.

These guidelines are most welcome. They afford an essential scientific, pragmatic and effective patient-centred resource for clinicians. They fill a critical gap in our response to HIV. Their sound professionalism and evidence-based wisdom will contribute to the policy shifts we critically need, if South Africa’s response to people who use drugs is to meet the standards of the Constitution.
